# A pilot study of the use of EEG-based synchronized Transcranial Magnetic Stimulation (sTMS) for treatment of Major Depression

**DOI:** 10.1186/1471-244X-14-13

**Published:** 2014-01-18

**Authors:** Yi Jin, Bill Phillips

**Affiliations:** 1 NeoSync, Inc., 4019 Westerly Pl., #201, Newport Beach, CA 92660, USA

**Keywords:** sTMS, MDD, rTMS, NEST, IAF, Depression

## Abstract

**Background:**

Repetitive Transcranial Magnetic Stimulation (rTMS) is an effective treatment for Major Depressive Disorder (MDD), and is based upon delivery of focal high-energy pulses of electromagnetic stimulation. We postulated that delivery of rTMS at the subject’s individual alpha frequency (synchronized TMS, or sTMS) would achieve efficacy with lower energy of stimulation. We developed a device that rotates neodymium cylindrical magnets at three locations along the midline above the subject’s scalp to impart low-energy, sinusoidal-waveform magnetic brain stimulation over a broad area, and performed this efficacy study.

**Method:**

Fifty-two subjects with MDD were enrolled in a randomized, sham controlled, double-blind treatment study (Trial Registration: NCT01683019). Forty-six subjects were included in the final analysis. Most subjects received concurrent antidepressant medications that remained unchanged during the study. Subjects were randomized to three treatment groups: 1) active sTMS with a fixed frequency at the subject’s alpha frequency; 2) active sTMS with a random stimulus frequency that varied between 8 Hz and 13 Hz; and, 3) sham sTMS. 20 half-hour sTMS sessions were administered 5 days per week for 4 weeks.

**Results:**

Subjects with either fixed or random frequency active sTMS had statistically significantly greater percentage reduction in depression severity compared to sham (48.5% vs. 19.3%, respectively; p = 0.001). No significant difference was found between fixed and random groups (p = 0.30). No significant side effects were reported.

**Conclusions:**

These results suggest that sTMS may be an effective treatment for MDD.

## Background

Traditional repetitive Transcranial Magnetic Stimulation (rTMS) directs high field strength magnetic pulses to a single brain location, most commonly the dorsolateral prefrontal cortex (DLPFC), as a treatment for Major Depressive Disorder (MDD) [1]. The study by O’Reardon [2], and more recently in the OPT-TMS trial by George [3], showed that this technique is an effective treatment for MDD. In both studies, rTMS stimulation was administered at 120% of the motor threshold at a frequency of 10 Hz. The treatment paradigm was identical for all subjects, regardless of their symptoms or characteristics of brain function.

The mechanism of action of rTMS to achieve relief of depressive symptoms remains incompletely understood. The immediate effect of rTMS pulses on brain function is the entrainment of cerebral oscillations to the frequency of stimulation [4-9]. Evidence suggests that rTMS may achieve therapeutic effectiveness through resetting of thalamocortical oscillators [10]. Repetitive entrainment of endogenous oscillations to a 10 Hz frequency of stimulation may facilitate the reemergence of intrinsic cerebral rhythms, thereby restoring normal brain function. Questions remain, however, as to the optimal stimulation parameters to achieve this resetting of oscillators. Stimulation variables that have not been systematically studied include stimulus intensity, frequency, and location. We hypothesized that it may be possible to improve the effectiveness of rTMS treatment for resetting cortical oscillators by synchronizing the rTMS pulses to the frequency of the patient’s individual alpha frequency (IAF), called synchronous TMS (sTMS) [10]. By synchronizing TMS to the IAF, it may be possible to use a low magnetic field strength sinusoidal waveform applied broadly across the brain, in contrast to the focal high strength magnetic field pulses traditionally utilized.

We report here on the first pilot trial of an experimental device designed to administer low-energy sinusoidal waveform sTMS to patients suffering from MDD. This device uses three neodymium permanent magnets that rotate at a programmed frequency or set of frequencies at or near the subject’s IAF, thereby imparting low energy stimulation broadly over the brain to entrain brain oscillations and potentially reset thalamocortical oscillators. This pilot study was designed to examine the feasibility of the technique, and an initial methodological validation of the possible efficacy of this new treatment modality.

## Methods

The aim of the clinical trial was to determine the efficacy of the sTMS treatment in comparison to sham, and to examine issues of safety related to the investigational device.

### Experimental device

**Figure 1:**
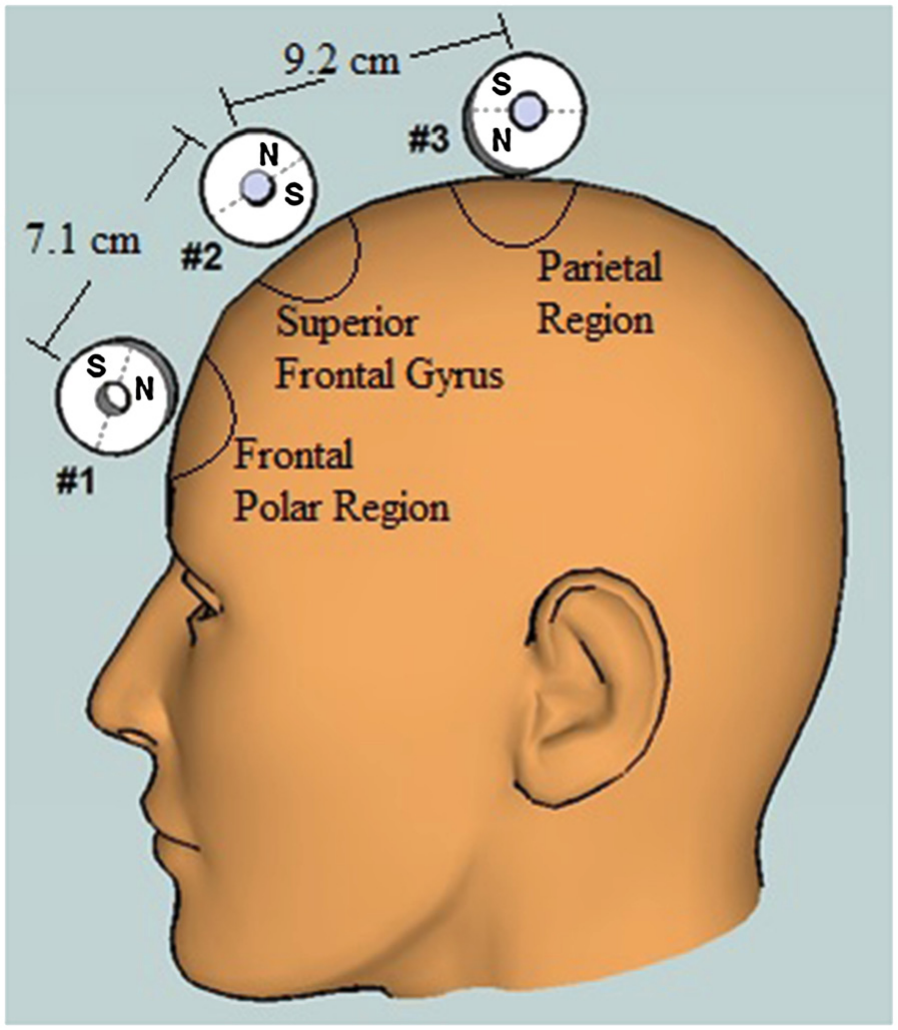
Location of the three cylindrical magnets above the subject’s head.

The sTMS device employs cylindrical neodymium magnets that are diametrically magnetized, with a surface field of 6,430 Gauss (0.64 T). The three magnets are positioned sagitally along the midline with the axis of rotation perpendicular to the midline (See Figure 1). Magnet #1 is located just above the eyebrows, closest to the front polar region of the brain. Magnet #2 is on the top of the head, 7 cm behind Magnet #1 approximately overlying the superior frontal gyrus. Magnet #3 is 9 cm behind Magnet #2 approximately overlying parietal region of the cortex. The magnets are positioned to provide a global magnetic field distributed broadly across the midline cortical surface.

The primary differences between the sTMS device used in the study and a standard rTMS system, other than the delivery mechanism are the magnetic field waveform (i.e., sinusoidal vs pulsed) and the intensity of stimulation. The maximum change in magnetic field over time (max dB/dt) for the sinusoidal waveform is 227 Gauss/msec, whereas the max for rTMS is approximately 185 kGauss/msec. Because the electric potential induced by a magnetic field is proportional to dB/dt, the energy of the sinusoidal magnetic field is estimated at less than 1% that of a standard rTMS system. Therefore, sTMS stimulation is sub-threshold and does not directly cause neuronal depolarization, but instead uses a low-level alternating induced electric field to entrain neuronal firing at the programmed frequency.

**Figure 2:**
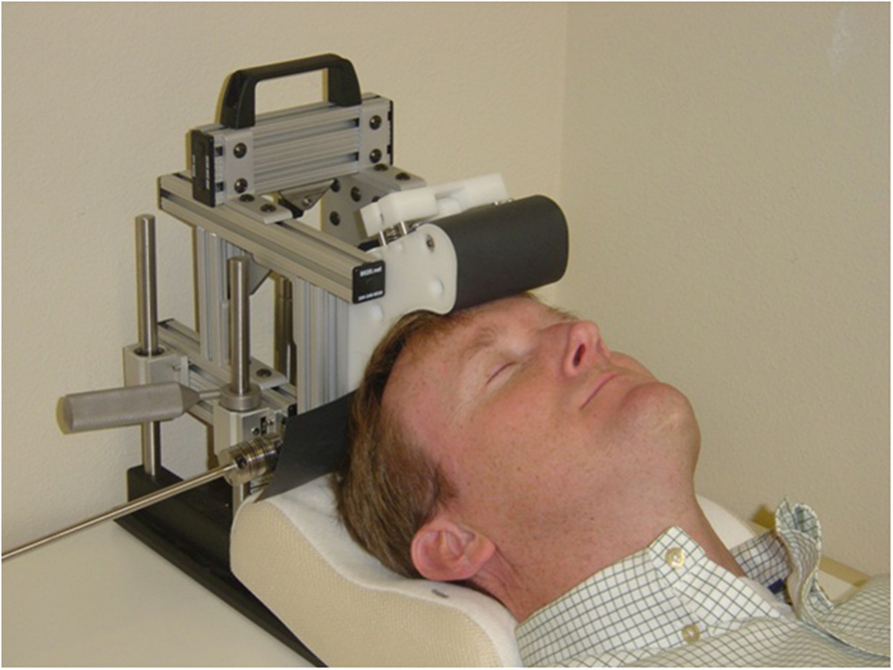
Subject lying under the magnet housing of the sTMS device undergoing treatment.

The device uses a mechanical closed-loop control system to rotate magnets secured inside an assembly. The magnet assembly is lowered and secured in place on the subject’s scalp. The subject lays face up under the device with eyes closed while the magnets rotated above the subject’s head (Figure 2).

To create a sham for a double-blinded trial, we constructed the sTMS device in the same way, except that it rotated non-magnetized steel cylinders instead of cylindrical magnets. The sham operated identically to the active device, except that no alternating magnetic field was generated. The sham was indistinguishable from the active device in that it looked identical, and had a similar sound and vibration when in operation.

### Procedures and subjects

#### Trial summary

This double-blinded, randomized, three-arm clinical trial was conducted at two sites in Newport Beach, California and Beijing, China. A total of 52 subjects with MDD were enrolled. All subjects were diagnosed by a physician as having moderate to severe MDD using DSM IV criteria based upon a structured clinical interview, with severity as defined by a Hamilton Depression Rating Scale (HAMD-17) [11] greater than or equal to a score of 17. Study subjects were treated five days per week for four weeks, with treatment consisting of a single 30-minute session. All treatment sessions were administered at the study site. Subjects were evaluated weekly throughout the trial to track progress and monitor any safety issues. All study subjects were allowed to use concomitant antidepressant treatment but were required to be stable on the medication for greater than one month prior to the start of the study. No adjustment of antidepressant medication was allowed during the study. The determination of clinical outcome was based on change in HAMD-17 score at the end of 4 weeks of treatment.

### Ethical approval

All experimental procedures were conducted with the approval of the appropriate IRB (Alpha IRB, San Clemente, CA and Peking University Hospital IRB, Beijing, China). The study was conducted with the understanding and written consent of each participant both in the US and China. The study was registered with clinicaltrials.gov (NCT01683019).

### Inclusion and exclusion criteria

#### Inclusion criteria

Subjects could be included only if they satisfied the following inclusion criteria: (1) 18 years of age or older; (2) diagnosis of MDD with HAM-D 17 ≥ 17; and (3) on a stable dose of the existing medication or no medication for 1 month or longer prior to the study.

#### Exclusion criteria

Subjects were excluded from study participation if any of the following exclusion criteria applied: (1) Diagnosed with another primary Axis I illness; (2) recent history of or current substance abuse; (3) clinically significant medical illness, including any thyroid disorders; or (4) known pregnancy and/or lactation, or intent to become pregnant during the study.

### Randomization

Each subject’s EEG was recorded after randomization to determine his or her IAF. EEGs were recorded using a Cadwell Easy II unit, and the IAF was found using a proprietary algorithm designed to detect the frequency in the alpha range (8.0 – 13.0 Hz) with maximum energy, confirmed through visual inspection of the EEG signal. Study subjects were randomized to one of three treatment arms with equal probability. The randomization table was created using a random number generator treating the all subjects as a single group. No blocking or stratification was used. The treatment arms were:

• Fixed frequency magnet rotation, set to the subject’s average IAF ± 0.1 Hz.

• Random frequency magnet rotation, which hopped to random frequencies between 8.0-13.0 Hz once per second, with a resolution to 0.1 Hz.

• Sham. Treatments appeared and sounded similar to active treatment except that no magnetic field was generated.

### Outcomes and data analyses

The primary objective of the study was to establish the efficacy of sTMS in comparison to a sham treatment in subjects with moderate to severe MDD. The treatment outcomes were analyzed for the intent-to-treat sample with the primary outcome being the percent reduction from baseline to endpoint on the HAMD-17 rating scale, using the last observation carried forward (LOCF) method for interpolating final score for those subjects who discontinued before the final endpoint. Non-parametric analysis was used to compare grouping differences as measured by the number of responders (defined as > 50% reduction from baseline to final endpoint) and remitters (defined as a final HAMD-17 ≤ 7). Analysis of Variance with repeated measure of time followed by post-hoc t-tests as indicated for each time point was performed to determine if there was as difference in outcome among the three groups based on percent reduction in symptom severity. The secondary objective was to describe the safety profile of the device in a clinical setting.

## Results and discussion

As shown in Table 1, no clinically relevant differences between the two sites were observed at baseline for demographic characteristics or baseline MDD severity. Table 2 lists all concomitant medications used by subjects in the study; there was no significant difference in the number or type of medications among subjects in the various response or treatment groups. The medications were not thought to affect the subjects’ alpha frequency, and therefore are not considered a factor.

**Table 1:** Clinical study demographic and baseline clinical information

	**Active sTMS**	**SHAM**	**Statistics**
**Gender (Male: Female)**	13:16	7:9	*X*^2^ = 0.11, p = 0.74
**Age (s.d.)**	42.5 (15.0)	46.3 (12.7)	*t* = 0.85, p = 0.40
**Years of education (s.d.)**	13.9 (4.2)	14.1 (3.2)	*t* = 0.16, p = 0.87
**Duration of illness in months (s.d.)**	11.1 (9.7)	13.6 (11.4)	t = 0.79, p = 0.44
**Hamilton score (s.d.)**	21.3 (4.0)	19.4 (4.1)	*t* = 1.55, p = 0.13
**Antidepressants (SSRI / other)**	28 /3	15 /0	*X*^2^ = 1.55, p = 0.21
**Alpha EEG frequency (s.d.)**	9.7 (0.9)	9.3 (1.1)	*t* = 1.39, p = 0.17

**Table 2:** List of concomitant medication used by subjects in the study, with the number of subjects using the medication

Alprazolam (4)	Esomeprazole (1)	Oxycodone (1)
Amitriptyline (3)	Fluoxetine (10)	Paroxetine (4)
Budesonide (1)	Flupentixol (4)	Pentosan Polysulfate (1)
Bupropion (3)	Fluvoxamine (1)	Sertraline (3)
Busparinone (1)	Gabapentin (1)	Sucralfate (1)
Cetirizine (1)	Lorazepam (5)	Sumatriptan (1)
chlorpromazine (1)	Maprotiline (1)	Tolterodine (1)
Citalopram (3)	Mentropolol (1)	Topiramate (1)
Clonazepam (6)	Metaxalone (1)	Venlafaxine (4)
Duloxetine (1)	Montelukast (1)	
Escitalopram (3)	Olazapine (1)	

Note, some subjects were on more than one medication.

Fifty-two subjects enrolled in the study. Six subjects withdrew in the first week due to problems with transportation to the study sites and one subject withdrew during Week 3. Data were analyzed using all subjects who completed at least one efficacy assessment (46 in total), with the last available HAMD-17 value carried forward for Week 4 outcome analysis (LOCF method).

No study site by treatment interaction was found (F_1,44_ = 0.22, p = 0.64), so that data were pooled across study sites. The HAMD-17 score mean and standard deviation at baseline and at the end of each week of treatment were determined, as shown in Table 3. For both active groups, no significant treatment difference between fixed frequency and random frequency was found (F_1,28_ = 1.09, p = 0.30). Therefore, the fixed and random frequency groups are combined into a single active group for this analysis.

**Table 3:** Weekly HAMD-17 scores for the three groups

	**SHAM**	**Fix**	**Random**
**Baseline (s.d.)**	20.0 (4.6)	21.4 (3.9)	20.8 (3.7)
**Week 1 (s.d)**	18.6 (5.4)	19.4 (3.6)	16.4 (7.1)
**Week 2 (s.d)**	17.8 (6.0)	17.0 (4.0)	14.6 (4.9)
**Week 3 (s.d)**	16.0 (5.3)	13.3 (5.1)	12.4 (4.4)
**Week 4 (s.d)**	15.9 (5.9)	11.7 (5.9)	10.1 (4.7)

**Figure 3:**
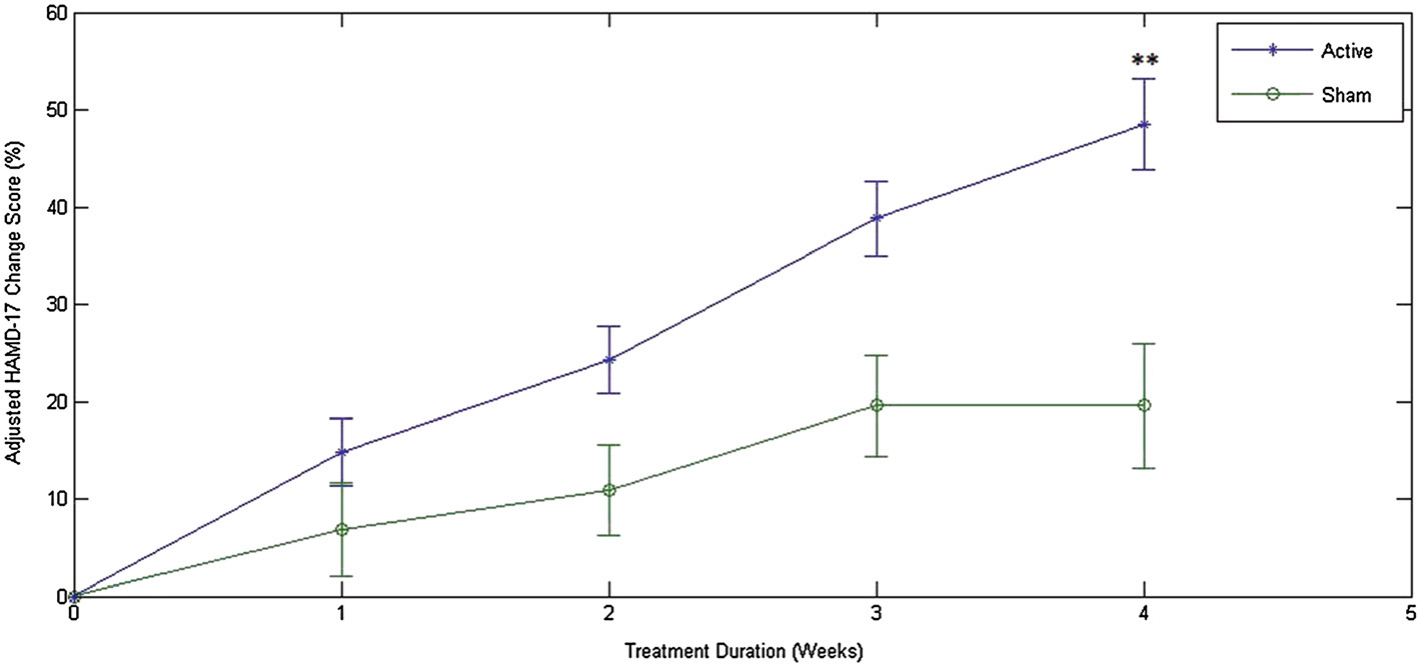
Average improvement in HAMD-17 score for the active group compared to sham (** shows p < 0.01).

The average percentage improvement in HAMD-17 score was determined for the active and sham groups, as shown in Figure 3. The active group was significantly better than the sham as an overall measure (F_1,44_ = 10.70, p = 0.002), and on a time-by-treatment interaction (Greenhouse-Geisser adjustment F_1.9,85.1_ = 4.1, p = 0.02). There were significantly more responders in the active treatment group (16/30 or 53.3%) compared to sham (2/16 or 12.5%) (χ^2^ = 7.30, p = 0.007). In the active group, 12/30 (37.9%) reached remission (HAMD-17 ≤ 7, six in the fixed frequency group and five in the random frequency group). Only one sham patient (6.25%) remitted (p = 0.015).

There was no significant change in IAF value during the 4 weeks of treatment. No subject reported a sensation during treatment other than feeling a slight vibration where the treatment arm made contact with the head. Approximately 50% of subjects (both sham and active groups) reported an occasional mild headache that went away shortly after treatment. This was likely due to vibrations of the prototype. A mild, light-headed feeling was reported by approximately 40% of subjects in the active group, either immediately or up to two hours after treatment. No sham subject reported light-headedness.

## Conclusions

In this study, a statistically significant decrease in HAMD-17 score was observed in subjects treated with the sTMS device compared to sham. These results indicate that a sub-threshold alternating sinusoidal magnetic field generated in the alpha frequency range can have therapeutic efficacy in patients with MDD. The sub-threshold alternating electrical field in the brain generated by the sTMS device would not actively depolarize neurons, but instead may influence neurons to fire at the induced frequency by modulating the threshold potential required for depolarization. These results support the hypothesis that a sinusoidal magnetic field applied at or near the IAF has a significant effect on brain function, possibly through entrainment of oscillations to the frequency of stimulation. This may help reset cortical oscillators, promote synaptic plasticity, and change brain function and patients’ mood through a mechanism similar to conventional rTMS systems [10].

The results of this study need to be interpreted within the context of several limitations. In the present study, clinical improvement was seen during the 4 weeks of treatment. It is not clear, however, that this is necessarily the optimal length of treatment. Subjects were contacted informally by telephone 4 weeks after the completion of the study to ensure there were no latent adverse effects, but no long-term clinical data was obtained. We would anticipate future studies to evaluate possible long-term effects of sTMS.

Our study found that the subjects treated at the IAF showed improvement that was numerically, but not statistically significantly greater, than subjects treated at an active random-frequency in the alpha range. These data suggest that there may be an advantage to matching the IAF, but neither prove nor disprove the hypothesis. One reason for this finding may be that the bandwidth of influence is large enough that any frequency in the alpha band may have an effect. This may also be a contributing factor to the efficacy of standard rTMS, which delivers treatment at a fixed 10 Hz, in the center of the alpha band. In a future clinical trial, it would be beneficial to compare the results of matching the IAF to an active device set to a more remote frequency, such as one in the beta or theta band.

The sTMS device used in this study was designed primarily to deliver stimulation at a specific frequency or set of frequencies. Other design decisions were made on a practical basis to make it feasible to build a first-generation sTMS device that could operate reliably in a multi-site clinical trial. Three rotating magnets were chosen to deliver treatment broadly over the midline region; it is possible that a different number of magnets in the same or different locations might match or exceed the efficacy seen in this study. As is evident in Figure 1, the rotating magnets are 180 degrees out of phase with each other. This creates the same phase relationship in the electric field generated in the brain. The relationship between the magnets was not chosen due to any perceived benefit of opposite phases. Instead, this relationship between magnets allowed for a smaller motor required to turn the magnets. Additional research into the benefits of different phase relationships would be valuable.

This study aimed to examine only the safety and efficacy of sTMS treatment. Future studies should also examine the effects of sTMS on various biological metrics, such as alpha power, frequency selectivity, blood flow, or EEG coherence. In the present study, both active groups (fixed and random frequency) had a positive effect on symptoms. Future studies should go further to establish clearly which technique achieves the most significant response. The present findings suggest that the sTMS device can be an efficacious treatment for MDD, and supports the conduct of a larger, definitive clinical trial.

## Abbreviations

ANOVA: Analysis of variance; DLPFC: Dorsolateral prefrontal cortex; EEG: Electroencephalogram; HAMD: Hamilton depression rating scale; IAF: Individual alpha frequency; LOCF: Last observation carried forward; MDD: Major depressive disorder; NEST: NeoSync EEG synchronized therapy; rTMS: repetitive TMS; sTMS: Synchronized TMS; TMS: Transcranial magnetic stimulation.

## Competing interests

All funding for this study was provided by NeoSync, Inc. Both authors were employed by NeoSync, Inc. at the time this study was performed. Both authors are minority shareholders in NeoSync, Inc. Both authors have patents pending related to the NEST device.

## Authors’ contributions

BP designed and managed manufacturing of the devices, maintained the devices during the study, and wrote the initial draft of the paper. YJ wrote the protocol, obtained IRB approval, managed the conduct at the study for both centers, and provided data analysis and critical review for the paper. Both authors read and approved the final manuscript.

## Authors’ information

Bill Phillips has over 10 years of experience in the medical device industry, primarily in the design and manufacture of implantable devices, such as ICDs and pacemakers. He received his PhD in Electrical and Biomedical Engineering from the University of Southern California. Primary research focused on functional brain mapping using EEG.

Yi Jin has over 25 years of experience in psychiatry and neuroscience, with a focus in clinical and electrophysiological research. He served as director of the Behavioral EEG laboratory of the Psychiatry Department at the University of California, Irvine. He is an executive board member of the EEG and Neuroscience Society. He completed his MD at Shanghai Medical University.

## Pre-publication history

The pre-publication history for this paper can be accessed here:

http://www.biomedcentral.com/1471-244X/14/13/prepub

## References

[B1] Price JL Drevets WC Neural circuits underlying the pathophysiology of mood disorders Trends Cogn Sci 2012 16 1 61 71 10.1016/j.tics.2011.12.011 22197477

[B2] O’Reardon JP Efficacy and safety of transcranial magnetic stimulation in the acute treatment of major depression: a multisite randomized controlled trial Biol Psychiatry 2007 62 11 1208 1216 10.1016/j.biopsych.2007.01.018 17573044

[B3] George MS Daily left prefrontal transcranial magnetic stimulation therapy for major depressive disorder: a sham-controlled randomized trial Arch Gen Psychiatry 2010 67 5 507 516 10.1001/archgenpsychiatry.2010.46 20439832

[B4] Thut G Rhythmic TMS causes local entrainment of natural oscillatory signatures Curr Biol 2011 21 14 1176 1185 10.1016/j.cub.2011.05.049 21723129 PMC3176892

[B5] Fuggetta G Pavone EF Fiaschi A Manganotti P Acute modulation of cortical oscillatory activities during short trains of high-frequency repetitive transcranial magnetic stimulation of the human motor cortex: a combined EEG and TMS study Hum Brain Mapp 2008 29 1 1 13 10.1002/hbm.20371 17318833 PMC6870897

[B6] Brignani D Manganotti P Rossini PM Miniussi C Modulation of cortical oscillatory activity during transcranial magnetic stimulation Hum Brain Mapp 2008 29 5 603 612 10.1002/hbm.20423 17557296 PMC6870908

[B7] Klimesch W Sauseng P Gerloff C Enhancing cognitive performance with repetitive transcranial magnetic stimulation at human individual alpha frequency Eur J Neurosci 2003 17 5 1129 1133 10.1046/j.1460-9568.2003.02517.x 12653991

[B8] Paus T Sipila PK Strafella AP Synchronization of neuronal activity in the human primary motor cortex by transcranial magnetic stimulation: an EEG study J Neurophysiol 2001 86 4 1983 1990 11600655 10.1152/jn.2001.86.4.1983

[B9] Veniero D Brignani D Thut G Miniussi C Alpha-generation as basic response-signature to transcranial magnetic stimulation (TMS) targeting the human resting motor cortex: a TMS/EEG co-registration study Psychophysiology 2011 48 10 1381 1389 10.1111/j.1469-8986.2011.01218.x 21542853

[B10] Andrew F Leuchter IA Cook YJ Phillips B The relationship between brain oscillatory activity and therapeutic effectiveness of transcranial magnetic stimulation in the treatment of major depressive disorder Front Hum Neurosci 2013 7 37 1 12 23550274 10.3389/fnhum.2013.00037 PMC3581824

[B11] Hamilton M “A rating scale for depression,” J Neurol Neurosurg Psychiatry 1960 23 56 62 10.1136/jnnp.23.1.56 14399272 PMC495331

